# Self-reporting on foot health status in children

**DOI:** 10.1186/1757-1146-8-S2-O29

**Published:** 2015-09-22

**Authors:** Stewart C Morrison, Jill Ferrari

**Affiliations:** 1 Human Motor Performance Group, School of Health, Sport and Bioscience, University of East London, E15 4LZ, UK

**Keywords:** Self-report, foot, paediatrics

## Background

Clinicians involved in the management of children with chronic conditions are encouraged to utilise self-report outcome measures to inform clinical management and ensure patient preferences are considered throughout care provision. The use of self-report measures in the paediatric patient is not without challenges and alternative parent/proxy responders are often considered more appropriate. Despite this, the validity of parent/proxy responders reporting on behalf of children can be challenged and further work evaluating concordance between children and parents/proxy raters is needed. Through use of a foot-specific patient reported outcome measure in girls with Turner syndrome, the aim of this study was to measure agreement between children and parent/proxy responders for self-reporting on foot health.

## Methods

A cross-sectional questionnaire-based survey was conducted in a sample of British girls aged 8–14 with Turner syndrome (TS) and history of foot and/or ankle problems. Ten girls with TS (median age 9.5 years) and a nominated parent/proxy responder (n = 10) were recruited. The Oxford Ankle Foot Questionnaire for Children was used to assess foot-related health status. Raw scores were calculated and converted into percentage scores across the three domains and footwear item. Agreement between parent and children domain scores was determined with the weighted Kappa.

## Results

Agreement between parent/proxy and child scores was substantial, with a total kappa coefficient (Kw) of 0.71. Substantial agreement was found for the physical domain (Kw= 0.77) and almost perfect agreement for the school and play domain (Kw = 0.86). The lowest agreement was found for the emotional domain (Kw 0.52) and footwear item (Kw 0.58) with moderate agreement for both.

## Conclusion

The findings from this study highlight a disparity between child and parent/proxy responders for some domains. This finding echoes previous research where agreement between responders was observed for the physical domain(s) or observable traits but not for emotional or social domains. The findings from this study offer some support for using self-report outcomes measures in the evaluation and clinical management of paediatric foot problems associated with chronic conditions.

